# Evaluation of new data processing algorithms for planar gated ventriculography (MUGA)

**DOI:** 10.1120/jacmp.v10i3.2977

**Published:** 2009-07-08

**Authors:** Joanna R. Fair, Philip H. Heintz, Robert J. Telepak

**Affiliations:** ^1^ Department of Radiology University of New Mexico School of Medicine Albuquerque New Mexico

**Keywords:** MUGA, automated data processing, radionuclide ventriculography, ejection fraction, left ventriculography, phantom, clinical testing, nuclear medicine

## Abstract

Before implementing one of two new LVEF radionuclide gated ventriculogram (MUGA) systems, the results from 312 consecutive parallel patient studies were evaluated. Each gamma‐camera acquisition was simultaneously processed by semi‐automatic Medasys Pinnacle and by fully automatic and semiautomatic Philips nuclear medicine computer systems. The Philips systems yielded LVEF results within ±5LVEF percentage points of the Medasys system in fewer than half of the studies. The remaining values were higher or lower than those from the long‐used Medasys system. These differences might have changed cancer patient chemotherapy clinical decisions. As a result, our institution elected not to implement either new system.

PACS: 87.57.U‐ Nuclear medicine imaging

## I. INTRODUCTION

The MUGA (multigated acquisition) study has long been used as a non‐invasive method for quantification of left ventricular ejection fraction (LVEF).^(^ ^1^ ^–^ ^3^ ^)^ One of the first such programs commercially implemented was developed over 30 years ago by the Medical Data Systems (MDS) company. The company later became Medasys and produced the Pinnacle MUGA system. While other imaging techniques such as quantitative gated SPECT (QGS) and volumetric ultrasound have subsequently been developed for LVEF determination, planar radionuclide ventriculography remains a generally accepted standard against which other LVEF measurement techniques are evaluated.^(^ ^4^ ^–^ ^8^ ^)^ In particular, at institutions with active cancer treatment centers, this study is frequently performed on patients receiving chemotherapy regimens with various cardiotoxic drugs.^(9)^ Accurate LVEF measurements are critical to safe initiation and continuation of chemotherapy. Precise cutoffs for normal LVEF vary among institutions and protocols. At our institution, a five percentage point decrease is generally considered to represent significant myocardial toxicity. Additionally, our institutionally accepted normal range for LVEF is 55%–75%. If the LVEF falls below the lower limit of 55%, treatment protocols recommend – and individual oncologists consider – discontinuing or delaying therapy.^(^ ^10^ ^–^ ^13^ ^)^

For over 17 years, the standard planar radionuclide ventriculogram at our institution had been the Medasys Pinnacle MUGA study. However, when Medasys went out of business several years ago and stopped supporting their hardware and software, our institution sought a replacement system. No universally accepted gold standard exists for left ventricular ejection fraction.^(14)^ As a surrogate for a gold standard, we used the long‐tested Medasys Pinnacle system as the reference standard for testing new software packages. As we already had a Philips Odyssey nuclear medicine computer system, we elected to test the Philips systems in parallel with our Medasys Pinnacle system by installing hardware to acquire data simultaneously into both systems. Before conducting this test, we verified that the Philips GBP‐Dual system had already been clinically validated in 30 patients by Philips. This test was done by comparison with Medasys Pinnacle; however, the results were never published (per telephone conversation between RJ Telepak and WR Carpentier of Scott and White Clinic, Temple, TX).

To understand why we decided to clinically test the two systems, it is important to discuss briefly how each processing algorithm works. The Medasys Pinnacle system is semiautomated: the operator positions a box defining the search limits for the algorithm to find the edges of the left ventricular (LV) chamber using standard second‐derivative search criteria. The operator employs Fourier phase data to help position this box accurately to exclude left atrial (LA) activity. The operator then visually evaluates the data to exclude right ventricular (RV) activity and non‐cardiac (e.g. splenic) activity from the area being searched. The sensitivity of the edge‐detection algorithm can be manually adjusted if necessary in the four quadrants of the LV chamber to maximize edge tracking accuracy. This system has demonstrated reproducible results at our institution for over 17 years and thus serves as a reasonable standard for testing the new algorithms.

At the onset of this project, Philips Medical Systems Odyssey systems had two available planar radionuclide ventriculography software packages; we elected to test both. The first system, the Philips GBP‐Dual system (GBP‐D), was written to emulate the Medasys program on the more modern Odyssey computer platform. One notable difference is that this system is fully automatic. As a result, the operator cannot define or limit the LV search area or adjust edge‐detection sensitivity. Likewise, Fourier phase data is available but cannot be used by the technologist to define the search area for the LV. The second system is the Philips GBP‐Multi software (GBP‐M) package. This software does not directly emulate the Medasys system, but its semiautomated algorithm permits the operator to define a search area for the LV.

Our goal was to determine how closely the left ventricular ejection fractions calculated by the newer systems reproduced the calculation of the Medasys system. In particular, we wanted to see how often discrepancies in the calculated LVEF might affect treatment decisions.

## II. MATERIALS AND METHODS

The study group included all patients who underwent gated planar left ventriculography (MUGA) at our institution in the two‐year period from May 2004 through April 2006. Three hundred and fifteen (315) total examinations were performed, including repeat examinations on 37 patients. Of these examinations, three examinations processed only with the Medasys system (due to technical difficulties) were excluded from the analysis.

Red blood cell labeling was performed *in vivo* with intravenous stannous pyrophosphate followed by Tc‐99m sodium pertechnetate (Cardinal Health). The patients were then imaged in the best LAO projection to separate RV and LV with a Siemens Orbiter gamma camera, which had already undergone acceptance testing. The raw data from the gamma camera were sent to the Medasys and Philips hardware simultaneously and processed by all three software systems independently. This simultaneous acquisition and processing method was the same as that employed by Carpentier for the original validation of the GBP‐D system. All three systems provided LVEF data for analysis; only the Medasys results were reported to clinicians for therapy decisions.

The data were then analyzed using a least‐squares fit comparing the Medasys LVEFs to the test systems’ LVEFs to determine degree of correlation, forcing the fit through (0,0). In all of the tests, the Medasys results were used as the basis for comparison. In addition, we performed a Bland‐Altman analysis^(15)^ of the differences between the Medasys and test systems versus the means of the paired values. The relevant clinical limits of ±5 ejection fraction percentage points difference between the test systems and Medasys were selected based on our institutional standard that such a difference represents a clinically significant change in LVEF. The mean difference and 95% confidence intervals were calculated to determine whether the test systems’ results generally fell within the clinic limits with respect to the Medasys results.

LVEF determination by Quantitative Gated SPECT (QGS) from stress sestamibi perfusion myocardial scans is known to cause false‐high measurements in patients with very small LV volumes.^(^ ^16^ ^–^ ^17^ ^)^ For this reason, we also collected data of LV end diastolic volume (LVEDV) to evaluate whether small volumes might contribute to different results between the Medasys and the test systems. Only Medasys and GBP‐D provided this data, not the GBP‐M system.

## III. RESULTS

**Figure 1:**
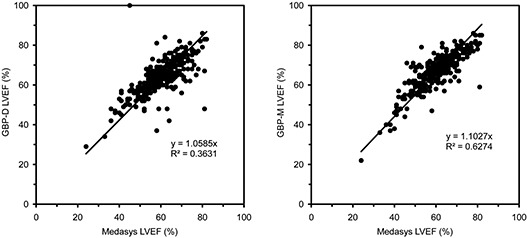
Plot of LVEFs calculated by: a) GBP‐D and b) GBP‐M systems versus the Medasys system. Superimposed is a least‐squares fit (forced through 0,0).

**Figure 2:**
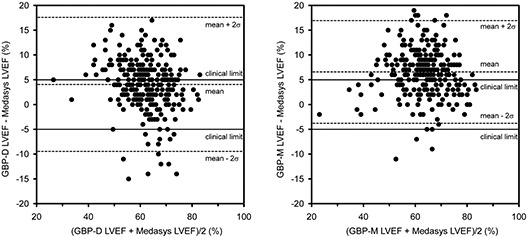
Bland‐Altman analysis of: a) GBP‐D and b) GBP‐M systems compared with the Medasys system; solid lines represent the clinical limits (i.e. ±5 ejection fraction percentage points), while the dashed lines represent the mean and 95% confidence intervals.

Figure 1 shows plots of (a) GBP‐D and (b) GBP‐M LVEFs versus the Medasys LVEFs. Linear least‐squares fits of both the test systems’ data relative to the Medasys data are also shown. These plots demonstrate relatively poor correlation between the test systems and the Medasys systems overall. It is important to note that there is a wide spread in both test systems’ data relative to the Medasys data, with LVEF values scattered both greater than and less than the Medasys LVEFs. The Bland‐Altman analyses in Fig. 2 show that for both test systems, the 95% confidence intervals fall well outside the clinical limits of ±5 ejection fraction percentage points. Overall, only 168 calculations (54%) of the GBP‐D system and 116 calculations (37%) of GBP‐M system agreed with Medasys to within ±5LVEF percentage points. Nineteen (7%) of the GBP‐D system and 4 (1%) of the GBP‐M system data were more than 5 percentage points lower, while 125 (40%) of the GBP‐D system and 192 (62%) of the GBP‐M system were more than 5 percentage points higher.

**Figure 3:**
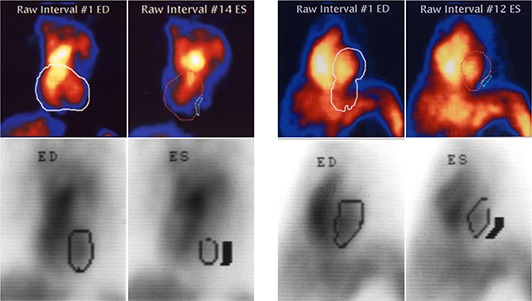
Two examples: (a and b) of left ventricular end diastolic volume (ED) and left ventricular end systolic volume (ES), respectively, determination by GBP‐D (color figures, top) versus Medasys (black‐and‐white figures, bottom).

These figures also demonstrate a number of cases with markedly different values of LVEF obtained by the test systems than by the Medasys system. We assessed these differences by visually inspecting the left ventricular contour determined by each algorithm. The cases with the worst correlation typically showed that the search algorithm had extended the boundaries well outside the visualized LV boundary. In particular, for the fully automated GBP‐D system, the search algorithm sometimes included one or more extraneous structures in addition to the LV, or entirely excluded the LV from the calculation. Figure 3 provides two such examples. In Fig. 3(a), the test system (top figures) included left ventricle and right ventricle in the LV area for end diastole (ED), while it included only the right ventricle in the LV area for end systole (ES). The bottom figures show that only the left ventricle is included by the Medasys system. Similarly, in Fig. 3(b), the test system included subdiaphragmatic activity from bowel or spleen in the LV area for end diastole (ED). The LV area for end systole (ES) did not include any specific extraneous structures but did extend well outside the visualized boundaries of the most intense counts (depicted in orange).

**Figure 4:**
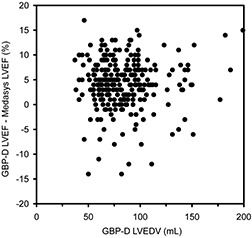
Difference between GBP‐D LVEF and Medasys LVEF versus GBP‐D LVEDV.

Unlike the QGS system of LVEF measurement, the LVEDV value for the GBP‐D system has no significant influence on LVEF measurements. As illustrated in Fig. 4, no correlation is seen between LVEDV and discrepancies between the GBP‐D and the Medasys LVEFs.

## IV. DISCUSSION

Our data indicate that the LVEFs calculated by the Medasys and GBP‐D methods lie within 5 LVEF percentage points in approximately half of the cases, with less agreement between the Medasys and GBP‐M systems. The remaining cases were significantly disparate, both higher and lower, with some GBP‐D and GBP‐M results markedly diverging from the Medasys calculation. These data show both a systematic trend – the test systems’ LVEFs were on average higher than those from the Medasys system – as well individual failures of the test systems in certain cases.

With regard to the systematic differences between the test systems and Medasys, it is difficult to determine which system correctly calculates the LVEF. First, there is no true gold standard for clinical determination of left ventricular ejection fraction, and therefore no definite reference technique.^(14)^ Additionally, although planar gated ventriculography (MUGA) has long been standard for calculation of left ventricular ejection fraction, many software packages have been produced for this purpose over the years.^(18)^ The need for validation of these packages was recognized early in the development of this technique, and both software phantoms and dynamic left ventricular phantoms have been employed for this purpose.^(^ ^19^ ^–^ ^20^ ^)^ In practice, however, many software programs are not extensively tested prior to clinical use. One of the most comprehensive and recent studies by De Bondt et al.,^(21)^ evaluated ten software packages already in clinical use against a dynamic left ventricular phantom with known ventricular volumes. Their results demonstrate that three of the ten software packages have systematic errors in LVEF determination. Of note, however, is the fact that the GBP‐M software tested in our study was included in the De Bondt study and did not demonstrate significant systematic errors. Additionally, their study showed no wide spread of the calculated GBP‐M LVEF values around the “real” LVEF values (directly obtained from the phantom). This contrasts with the wide spread in LVEF values calculated by this same software package in our study on real patients.

Without detailed access to manufacturers’ processing algorithms, it is not possible to determine precisely which features of the algorithm contribute to the discrepancies. One likely source of systematic difference observed in our study may be differences in background subtraction: relative oversubtraction of background counts elevates the calculated LVEF, while undersubtraction decreases it.

Such systematic differences could be identified by testing all new LVEF algorithms with a validated left ventricular phantom; however, such phantoms are not widely or commercially available. In the particular case of planar radionuclide ventriculography (MUGA), phantom studies present a challenge because a dynamic phantom (pumping radiopharmaceutical through a simulated contracting left ventricle) is not commercially available. Individual centers may not have access to such a phantom to do this testing. This type of testing may be best accomplished by manufacturers working with institutions with access to such a phantom.

More troubling than the overall systematic differences were the marked differences between some of the LVEFs calculated by the test programs and those calculated in the Medasys system. The observed variability may stem in part from significant patient variables in this patient population. Body habitus, difficulties in patient positioning, and medicines that interfere with *in vivo* RBC labeling may influence determination of LVEF in real patients compared to mechanical phantoms. In particular, such variability in real patients may overwhelm the capabilities of the left ventricular edge‐detection algorithms. Importantly, the phantom designed by De Bondt, modeled only the left ventricle. The marked variability observed in our study was in some cases attributable to inclusion of erroneous structures in the test systems’ left ventricular volume calculation, which cannot happen in this phantom.

To identify errors that arise in live patients, pre‐market validation of new algorithms should include testing in an adequate number of live patients, in addition to testing in phantoms. Our study in over 300 patients suggests that the initial clinical testing on 30 patients may not have adequately sampled the variabilities that live patients present. Additionally, smaller centers may not have adequate case volumes, experience, trained nuclear medicine specialists, or medical physicists necessary to do clinical testing on large numbers of patients. This effectively puts the onus of doing such testing on the manufacturers, likely in conjunction with a large institution.

As a result of this study, our institution decided not to implement these new software programs. The manufacturer was informed of our findings and pulled the product from the market. They further used our test data for validation of a new product now being sold.

## V. CONCLUSIONS

This study illustrates the importance of a large‐scale clinical evaluation of any new medical imaging tool that generates quantitative clinical values important to patient care. In this particular case, one of the newer software packages introduced fully automated functions to generate these values. Before purchasing a new system, we recommend seeking out all previous clinical evaluations of the equipment. If no such study has been performed, then the purchasing institution should strongly consider conducting such a study, particularly in comparison with a clinically tested system.

More generally, our results suggest that adequate testing of new software packages should include phantom studies, when possible, and clinical testing on a reasonable number of patients.
